# Whole-brain white matter correlates of personality profiles predictive of subjective well-being

**DOI:** 10.1038/s41598-022-08686-z

**Published:** 2022-03-16

**Authors:** Raviteja Kotikalapudi, Mihai Dricu, Dominik Andreas Moser, Tatjana Aue

**Affiliations:** Institute of Psychology, University of Bern, Fabrikstrasse 8, 3012 Bern, Switzerland

**Keywords:** Cognitive neuroscience, Emotion, Social behaviour, Social neuroscience

## Abstract

We investigated the white matter correlates of personality profiles predictive of subjective well-being. Using principal component analysis to first determine the possible personality profiles onto which core personality measures would load, we subsequently searched for whole-brain white matter correlations with these profiles. We found three personality profiles that correlated with the integrity of white matter tracts. The correlates of an “optimistic” personality profile suggest (a) an intricate network for self-referential processing that helps regulate negative affect and maintain a positive outlook on life, (b) a sustained capacity for visually tracking rewards in the environment and (c) a motor readiness to act upon the conviction that desired rewards are imminent. The correlates of a “short-term approach behavior” profile was indicative of minimal loss of integrity in white matter tracts supportive of lifting certain behavioral barriers, possibly allowing individuals to act more outgoing and carefree in approaching people and rewards. Lastly, a “long-term approach behavior” profile’s association with white matter tracts suggests lowered sensitivity to transient updates of stimulus-based associations of rewards and setbacks, thus facilitating the successful long-term pursuit of goals. Together, our findings yield convincing evidence that subjective well-being has its manifestations in the brain.

## Introduction

What makes one experience more frequent positive feelings, become better adjusted socially and stay in good mental and physical health? A large body of research has shown that subjective well-being, which includes perceived mental and physical health as well as rewarding social interactions^1^, has less to do with objective circumstances such as age, gender, race, income, education or marital status and more to do with stable personality traits^2–5^. Although the effects of outside circumstances on well-being are often statistically weak^6–8^, people persist in their impression that factors such as a relocation, a higher income, getting married or having children would lead to a substantial increase in subjective well-being^9–11^. This perceived importance of outside circumstances is likely the result of a focusing illusion^12–14^, i.e., the tendency to focus on the changes that singular events bring while overlooking various other factors that will eventually also influence the actual feeling states. This is of course not to say that situational factors are not influential in subjective well-being.

The current understanding of well-being^15–18^ is that genes (temperament) and personality together dictate a baseline of happiness. There is a range of values by which this baseline can fluctuate (i.e. a dynamic equilibrium;^16^), and this is where external events^19,20^, an individual’s goals and values^21,22^ as well as social comparisons with peers^23–25^ can exert most of their influence. Consequently, it is the interaction between genes and personality, on one hand, and situational factors and their interpretations, on the other hand, that give a complete picture to understanding well-being. The current study investigates the existence of well-being correlates in brain anatomy. Because we cannot study variations in brain anatomy for the person × situation interaction in total (i.e., across a multitude of different situations), and because the situation factor alone cannot be expected to manifest in brain anatomy, we concentrate on the person factor here.

Among personality factors that influence subjective well-being, the most researched ones have been a dispositional optimistic outlook on life^26–28^, a particular pattern of Big Five personality dimensions (i.e. high extraversion and low neuroticism;^29–32^), higher dispositional sensitivity to rewards than to setbacks (i.e., a high aroused behavioral activation (BAS) and/or a lowly aroused behavioral inhibition system (BIS);^33–36^) as well as the habitual use of particular emotion regulation strategies (i.e. a frequent use of cognitive reappraisal and/or a rare use of suppression of negative affect;^37–39^). These personality factors not only independently predict subjective well-being but they also share significant variance with each other (e.g. Big Five with cognitive reappraisal^40–43^ and with BIS/BAS^44^; optimism with Big Five^45–48^ and with cognitive reappraisal^49^; BAS with use of cognitive reappraisal and/or low suppression of negative emotions^35,50,51^). The question then arises of how exactly these personality factors position themselves in relation to each other and in their predictive power over subjective well-being. Our study aimed to investigate this by running a principal component analysis (PCA) that revealed the underlying commonalities between these psychological constructs.

One of the objectives of neuroscience is to map brain-behavioral connections which may yield important insights about the processes that possibly underlie psychological constructs. Several studies exist already on the gray and white matter correlates of personality measures^52–56^ and of subjective well-being^57^. However, a limitation of these studies is that they have focused on one personality measure at a time (see^58^). A likely possibility is that some personality measures cluster more often together than others, giving rise to personality “profiles” or “types”^59–61^. For example, high extraversion and high conscientiousness tend to cluster together but are both separate from high neuroticism^60,62^. Therefore, it might be wiser to investigate the neural correlates of personality profiles instead of individual factors. This has only very recently been attempted. For example, Li et al.^63^ have looked into the grey matter correlates of personality profiles. However, no study to our knowledge has investigated the white matter correlates of personality profiles, particularly profiles that predict subjective well-being.

White matter tracts connect various grey matter regions and their integrity is a marker of how well the corresponding grey matter regions communicate with each other (e.g.,^55^). Therefore, individual variability in white matter tracts may underlie variability in personality profiles. Accordingly, in our study, we correlated the “positive personality profiles” from the PCA with the markers of white matter integrity. Because no other study has investigated the white matter correlates of personality profiles that are predictive of subjective well-being, we based our hypotheses on the few studies that independently probed relevant concepts (i.e., Big Five, BIS/BAS, emotion regulation strategies) in relation to white matter integrity.

For example, several studies have linked neuroticism to decreased integrity of white matter tracts in the cingulum (CNG^56,64^), the uncinate fasciculus (UNC;^56,65^), the body of the corpus callosum (CC^66,67^) as well as increased integrity of the posterior CC, the corticospinal tract (CST), the inferior fronto-occipital fasciculus (IFOF;^68^) and the inferior longitudinal fasciculus (ILF;^67,68^). Extraversion correlates negatively with white matter integrity in the posterior CC^67,69^ and the CNG and the IFOF^69^. Furthermore, the BIS scale correlates positively with the integrity of the CNG, UNC, IFOF and superior longitudinal fasciculus (SLF;^68^). We note that some other studies have failed to find a meaningful relationship between white matter integrity and the Big Five dimensions^54,58,70^ or the BIS/BAS^71^. By contrast, the association between the habitual use of cognitive reappraisal and white matter integrity has been rather consistent, implicating the UNC^72–75^ and the CNG^72,73^. Given the above-reported correlates for isolated personality characteristics, we expected the implication of these areas in our profile study, too.

To measure white matter tracts, we used diffusion tensor imaging (DTI) tract-based spatial statistics, which is a commonly used voxel-based statistical framework for understanding white matter integrity^76^. To operationalize white matter integrity, we looked at widely used measures in DTI: (a) fractional anisotropy (FA), a measure that describes the directionality strength of local white matter tracts, (b) axial diffusivity (AD), a measure of the degree of diffusion of water molecules parallel to the axonal tracts, and (c) radial diffusivity (RD), a mean diffusion coefficient of water molecules diffusing orthogonally to the axonal tracts. These diffusivity measures provide complementary information about white matter integrity: as FA and AD values increase (integrity increases), RD values decrease.

## Results

### Principal component analysis results

**Figure 1:**
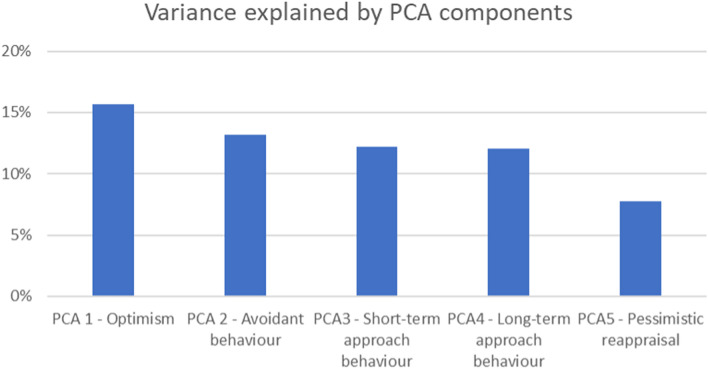
Total variance explained (60.8%) by the five PCA components with an eigenvalue > 1.

The PCA analysis revealed that the 16 behavioral measures loaded on five principal components (eigenvalue > 1, serving as a cutoff) (Table 1). Together, these five principal components or personality profiles explained 60.8% of the total variance (Fig. 1).

**Table 1:** PCA results derived from the questionnaire data.

Behavior data			Personality profiles derived from PCA				
Psychological constructs	Mean	Standard deviation	PCA 1 Optimism	PCA 2 Avoidant behavior	PCA 3 Short-term approach behavior	PCA 4 Long-term approach behavior	PCA 5 Pessimistic reappraisal
LOT optimism	8.71	2.08	**0.77**	− 0.24	0.05	0.04	0.02
LOT pessimism	4.04	2.02	− **0.69**	0.08	− 0.23	− 0.07	− 0.17
Satisfaction with life	26.51	5.33	**0.70**	− 0.02	0.25	0.11	− 0.05
COS optimism	69.94	11.01	**0.48**	− 0.12	− 0.31	0.30	− 0.01
COS pessimism	76.99	14.50	0.00	0.00	− 0.03	− 0.12	**0.78**
BIS	20.01	3.64	− 0.10	**0.80**	0.04	0.04	0.08
BAS drive	12.34	2.10	0.17	0.00	− 0.01	**0.73**	− 0.08
BAS fun seeking	12.26	1.63	− 0.11	− **0.39**	**0.41**	**0.41**	0.33
BAS reward response	16.59	2.04	0.08	0.00	0.18	**0.78**	− 0.08
BFI openness	7.49	1.88	− 0.27	− 0.31	0.25	**0.55**	0.14
BFI Conscientiousness	7.16	1.77	0.17	**0.44**	− 0.01	0.26	− 0.11
BFI extraversion	6.89	1.98	0.12	0.03	**0.75**	0.21	− 0.13
BFI agreeableness	7.05	1.33	0.15	0.11	**0.64**	− 0.13	0.11
BFI neuroticism	5.79	1.88	− 0.16	**0.82**	0.03	0.00	0.00
ERQ reappraisal	29.34	5.65	0.18	0.13	0.09	0.29	**0.51**
ERQ suppression	13.45	4.75	− 0.13	− 0.03	** − 0.69**	− 0.03	− 0.01

Descriptive statistics of mean and standard deviation of the behavioral questionnaires are presented. Bend correlations ‘rho’ between derived components and individual questionnaires are also provided. Bold indicates significant correlations (alpha = 0.05, corrected for multiple comparisons).

The first component, which we call “*Optimism*” loaded the LOT Optimism, COS Optimism and Satisfaction with Life positively and LOT Pessimism negatively. The second component, dubbed “*Avoidant behavior*”, loaded the BIS, BFI Conscientiousness and BFI Neuroticism positively and BAS Fun Seeking negatively. The third component, “*Short-term approach behavior*” loaded BFI Extraversion, BFI Agreeableness and BAS Fun Seeking positively but ERQ Suppression negatively. The fourth component, “*Long-term approach behavior*”, loaded all three BAS components and BFI Openness positively. Finally, the fifth component *“Pessimistic reappraisal”* loaded COS Pessimism and ERQ Reappraisal positively (Table 1).

### White matter voxel-based results

**Figure 2:**
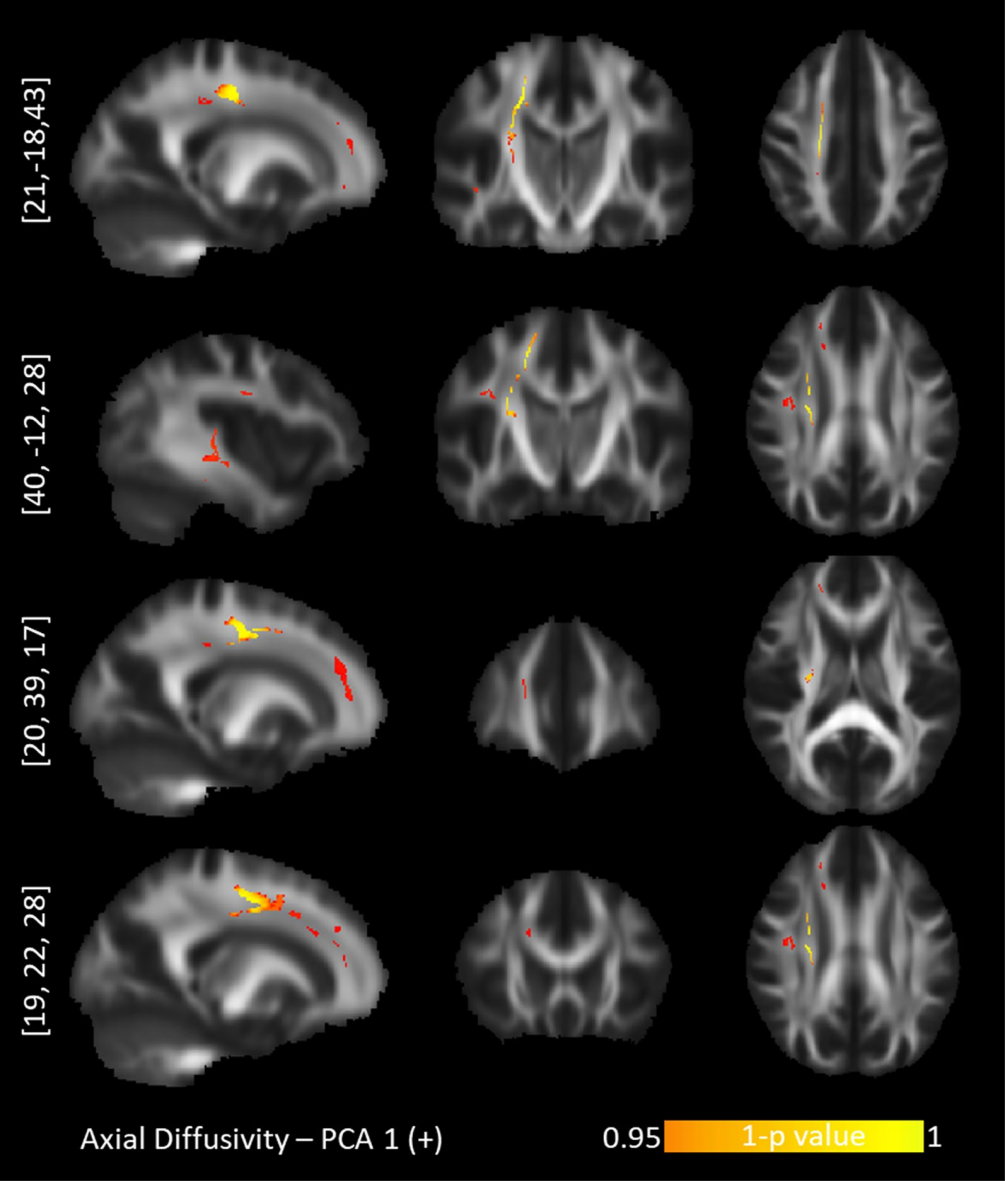
TBSS positive correlations between axial diffusivity and the "Optimism" personality profile. Upper row: corticospinal tract. Second row: dorsal superior longitudinal fasciculus. Third row: anterior corpus callosum. Bottom row: anterior cingulum bundle.

**Figure 3:**
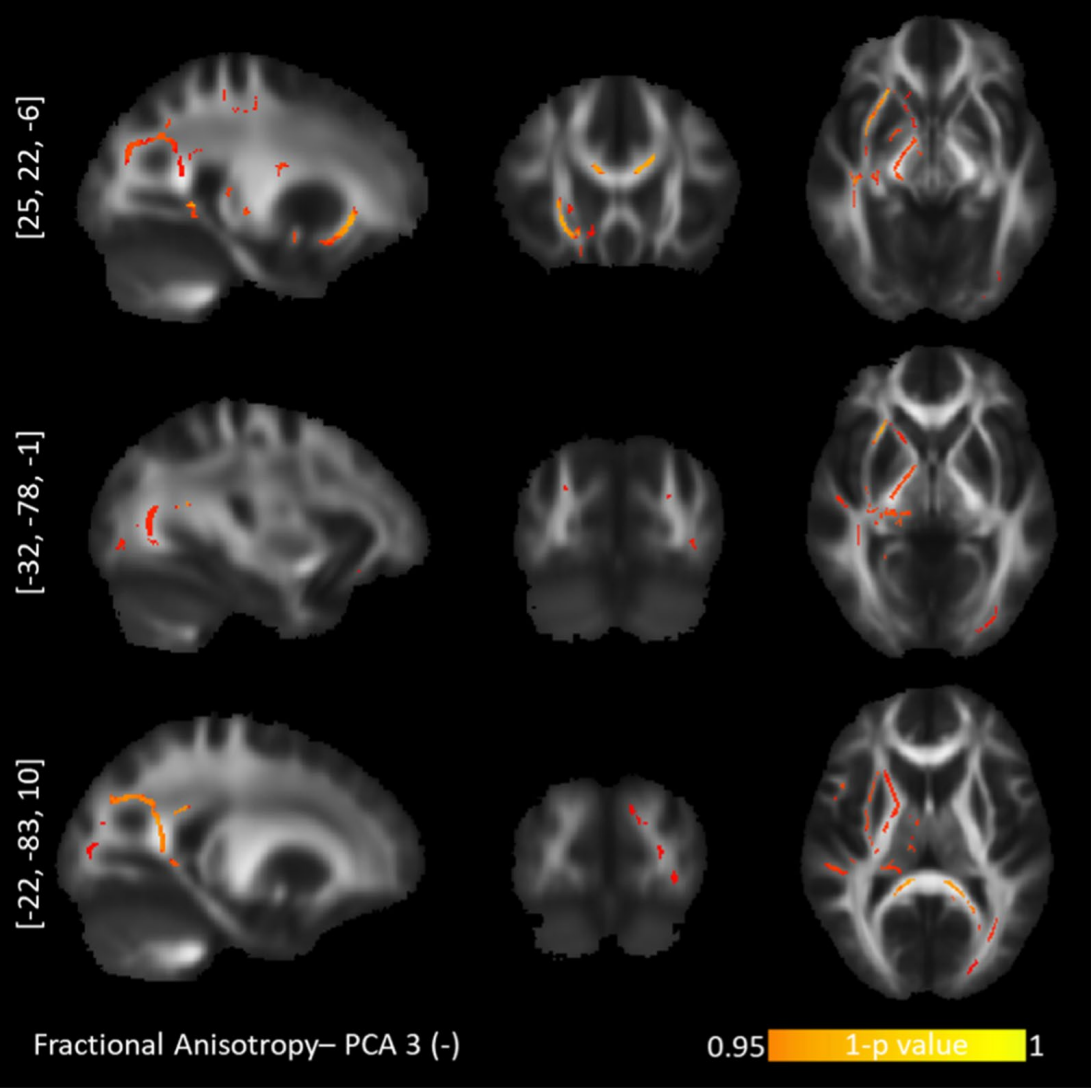
TBSS negative correlations between fractional anisotropy and the "Short-term approach behavior" personality profile. Upper row: inferior fronto-occipital fasciculus. Middle row: inferior longitudinal fasciculus/posterior corpus callosum. Bottom row: inferior longitudinal fasciculus/posterior corpus callosum.

**Figure 4:**
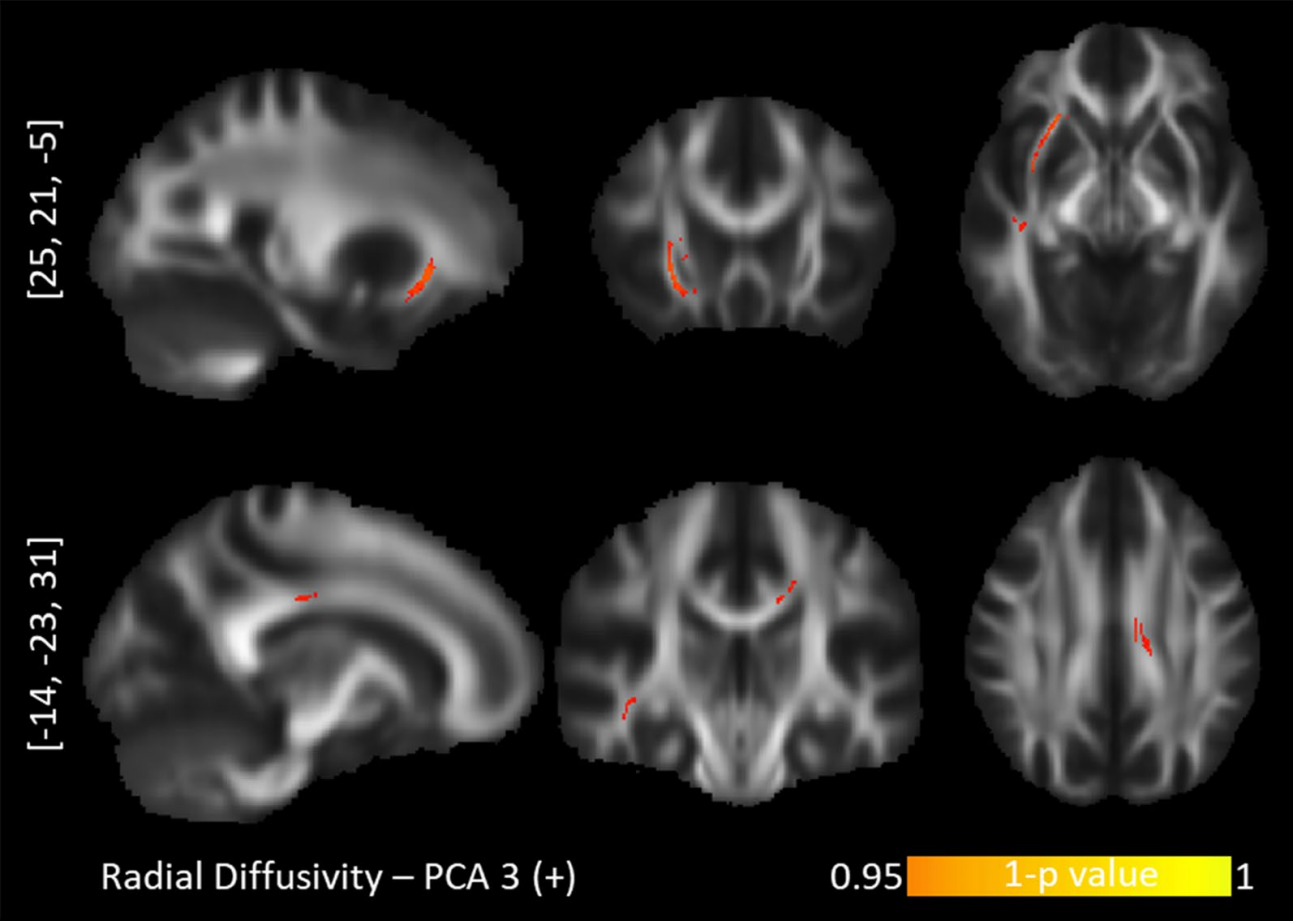
TBSS positive correlations between radial diffusivity and the "Short-term approach behavior". Top row: inferior fronto-occipital fasciculus. Bottom row: posterior corpus callosum.

**Figure 5:**
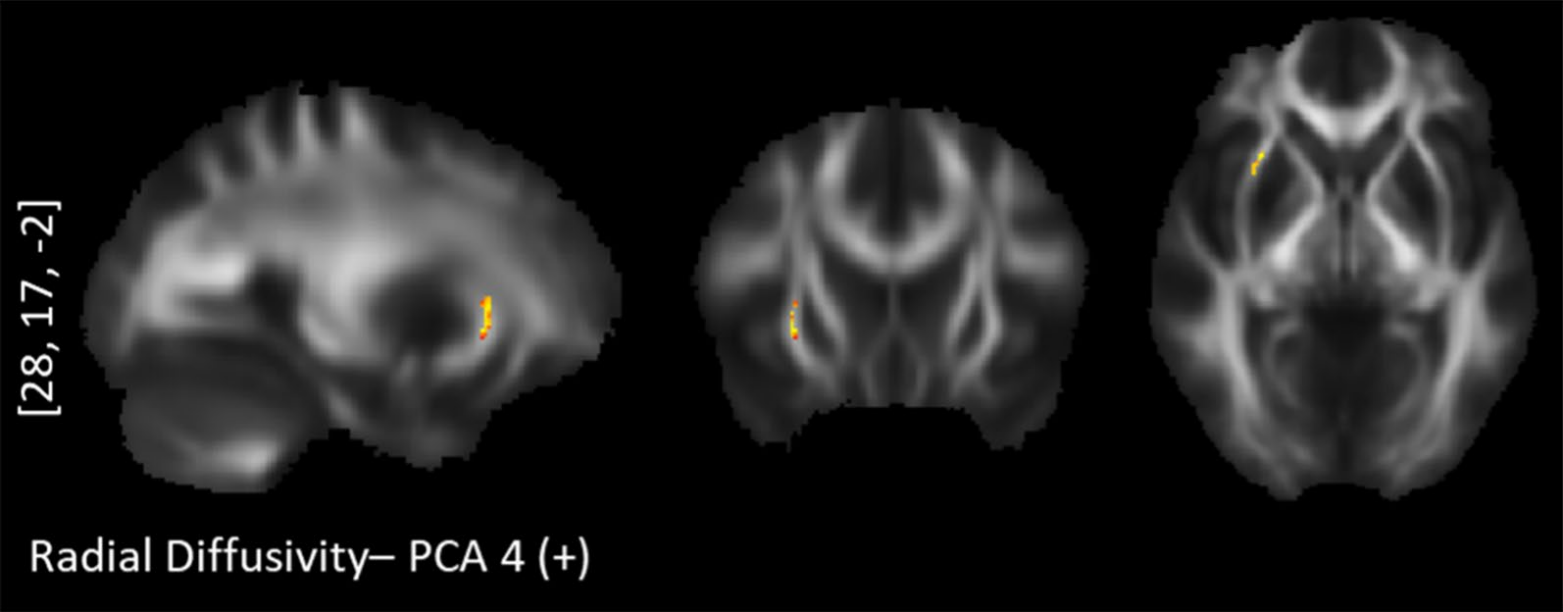
TBSS positive correlations between radial diffusivity and "Long-term approach behavor": right uncinate fasciculus.

The tract-based spatial statistics correlations between personality profiles and diffusion maps are presented with Table 2. Of these five PCA components, “*Optimism*” (PCA 1), “*Short-term approach behavior*” (PCA 3) and “*Long-term approach behavior*” (PCA 4) revealed tract-based findings for the probabilistic diffusion maps. Specifically, the “*Optimism*” component correlated positively with AD values in the right hemisphere exclusively (Fig. 2): the corticospinal tract (CST), the superior longitudinal fasciculus (SLF), anterior cingulum bundle (CNG), anterior corpus callosum (CC), anterior thalamic radiation (ATR) and uncinate fasciculus (UNC). The component for “*Short-term approach behavior*” correlated negatively with the FA values in the left hemisphere exclusively, namely the splenium of the CC, inferior longitudinal fasciculus (ILF), and UNC (Fig. 3). This component further correlated positively with the RD values in the right UNC/inferior fronto-occipital fasciculus (IFOF) and right SLF and left CNG (Fig. 4). Lastly, the component for “*Long-term approach behavior*” correlated positively with the RD values in the right UNC (Fig. 5).

**Table 2:** TBSS randomized results with TFCE at *p* < .05 after 5000 permutations.

Behavioral component	DTI marker	Cluster size	Peak voxel	Anatomy	Projections to gray matter regions
**Positive correlation with diffusion maps:** **PC 1—Optimism** 1. LOT (+) 2. SWLS (+) 3. COS optimism (+) 4. LOT pessimism (−)	AD	1781	[21, − 18, 43]	R corticospinal tract 72.5% R dorsal superior longitudinal fasciculus 33.7%	From primary motor and primary somatosensory cortex to afferent nerves in the body^106,184^
		102	[17, 20, 39]	R anterior corpus callosum 100% R anterior thalamic radiation 100% R anterior cingulum bundle 92.2%	Connecting the ventromedial prefrontal cortex, anterior cingulate cortex and frontal poles^135,136,185,186^
		81	[40, − 12, 28]	R dorsal superior longitudinal fasciculus 100% R corticospinal tract 50.6%	From angular gyrus to premotor cortices and caudal dorsolateral prefrontal cortex^105,106^
		70	[27, 35, − 1]	R anterior thalamic radiation 100% R rostrum of corpus callosum 92.86% R uncinate fasciculus 100%	Ventromedial prefrontal cortex and orbitofrontal cortex^135,136,187^
		32	[19, 22, 28]	R anterior cingulum bundle 100%	Anterior cingulate cortex and ventromedial prefrontal cortex^135,136^
		21	[6, 12, 31]	R ventral superior longitudinal fasciculus 100%	From supramarginal gyrus to ventral premotor cortex and pars opercularis^105,106^
**Positive correlation with diffusion maps:** **PC 3—Short-term approach behavior** 1. BFI Extraversion (+) 2. BFI agreeableness (+) 3. BAS fun seeking (+) 4. ERQ Suppression (−)	RD	905	[25, 21, − 5]	R Inferior frontal-occipital fasciculus 86.3% R Uncinate fasciculus	To ventromedial prefrontal cortex, orbitofrontal cortex^188–190^
		196	[− 14, − 23, 31]	L splenium of corpus callosum 100%	Posterior cingulate cortex and precuneus^135,136^
		31	[45, − 18, 37]	R superior longitudinal fasciculus II 96.8%	The premotor cortices, supplementary motor areas, pars opercularis^106^
**Negative correlation with diffusion maps:** **PC 3—Short-term approach behavior** 1. BFI Extraversion (+) 2. BFI agreeableness (+) 3. BAS fun seeking (+) 4. ERQ Suppression (−)	FA	7602	[25, 22, − 6]	R Inferior frontal-occipital fasciculus	
		6960	[− 10, − 17, 29]	L splenium of corpus callosum 49.6%	Posterior cingulate cortex^135,187^
		124	[− 32, − 78, − 1]	L splenium of corpus callosum 93.5% L inferior longitudinal fasciculus 100%	Medial parietal, medial occipital^135,187^
		85	[− 13, 16, 51]	L Corpus callosum (body)	Parietal to precentral regions^135,136^
		83	[− 22, − 83, 10]	L splenium of corpus callosum 100% L inferior longitudinal fasciculus 100%	Medial parietal, medial occipital^135,187^
		56	[− 11, − 86, 23]	**L** splenium of corpus callosum 100% L inferior longitudinal fasciculus 42.9%	Medial parietal, medial occipital^135,187^
		25	[− 36, 23, − 12]	L uncinate fasciculus 36%	Ventromedial prefrontal cortex, orbitofrontal cortex, inferior frontal cortex^188–190^
		20	[− 24, 13, − 13]	L uncinate fasciculus 100%	
		15	[− 19, − 80, 19]	L splenium of corpus callosum 100% L inferior longitudinal fasciculus 100%	Medial parietal, medial occipital^135,187^
**Positive correlation with diffusion maps:** **PC 4—Long-term approach behavior** 1. BAS Drive (+) 2. BAS Reward (+) 3. BAS Fun seeking (+) 4. BFI Openness (+)	RD	97	[28, 17, − 2]	R uncinate fasciculus 100% R Inferior frontal-occipital fasciculus	From anterior temporal lobes (via internal and external capsules) to inferior frontal cortex, vnPFC, orbitofrontal cortex, frontal pole^188–190^

The correlation results for behavior data with DTI maps for axial diffusivity (AD), radial diffusivity (RD) and fractional anisotropy (FA) are presented along with the cluster size, peak voxel coordinate and tract anatomy of the findings. LOT***—***Life LOT***—***Life Orientation Test. SWLS***—***Satisfaction With Life Scale. COS***—***Comparative Optimism Scale. BIS***—***Behavioral Inhibition System. BAS***—***Behavioral Approach System. BFI***—***Big Five Inventory. A plus (minus) sign depicts positive (negative) contributions of the behavioral measures to the principal components.

## Discussion

Our study is the first to investigate the white matter correlates of personality profiles that are predictive of subjective well-being. We used PCA to determine the possible profiles onto which dispositional optimism, Big Five dimensions, BIS/BAS, ERQ and SWL would load. We then searched for whole-brain white matter correlations with these profiles, and we based our hypotheses on past structural findings for each separate psychological construct. The PCA revealed five distinct profiles, three of which correlated to white matter measures: (a) “*Optimism*” profile, onto which LOT Optimism, COS optimism and SWLS loaded positively and LOT Pessimism loaded negatively; (b) “*Short-term approach behavior*” profile, onto which Extraversion, Agreeableness and BAS Fun Seeking loaded positively and EQ Suppression loaded negatively; (c) “*Long-term approach behavior*” profile, onto which BAS Drive, BAS Reward Responsiveness and, to a lesser degree, BAS Fun Seeking and Openness loaded.

We found that the “*Optimism*” profile correlated positively with AD values in white matter tracts exclusively in the right hemisphere: the CST, the SLF, and the anterior CNG, CC and ATR. Similar to FA values, the AD reflects the magnitude of diffusion parallel to fiber tracts and thus the integrity of axons^77,78^. The exclusivity of our findings in the right hemisphere is in line with the proposed functional asymmetry between the left and right hemispheres concerning complementary modes of processing incoming information (e.g.^79,80^). While the left hemisphere processes the information in a strictly sequential and unambiguously understood monosemantic context^81^, the function of the right hemisphere is to simultaneously capture a vast number of possible connections and to integrate them in an ambiguous but polysemantic context^81–83^. Because of this functionality, the right hemisphere has been involved in a variety of integrative phenomena^84–86^, including helping the implicit self^87^ maintain a positivity bias^88–90^. The implicit self is a highly integrative construct that processes vast amounts of self-relevant information from cognitive (e.g., autobiographical memories), motivational (e.g., values, needs) and affective (e.g., emotions) systems in parallel^87,91^. Because personal experiences are inherently perceived as positive in healthy individuals^92^, a functional implicit self is crucial in maintaining a positivity bias by assimilating negative affective experiences within a network of predominantly positive experiences^81,93^. Due to this holistic nature, the implicit self is believed to be a function of the right hemisphere^87,90^. The incapacity to regulate negative affect and display a (somewhat normal) positivity bias underlies emotional disorders such as depression, alexithymia and suicidal ideation, which are characterized both by a lack of optimism bias^53^ and a functionally deficient right hemisphere^79,81,94^, including decreased integrity of white matter tracts^95^. Our study thus supports previous findings that the right hemisphere is necessary for healthy individuals to express their positivity bias.

Regarding the individual tracts in the right hemisphere underlying “*Optimism*”, the CST was the strongest finding in terms of cluster size. The integrity of the CST is associated with a lower baseline excitability threshold, i.e. the minimum intensity at which the motor system can evoke a motor response^96,97^. At the same time, CST excitability indexes the degree of “wanting” a reward^98^; see also^99^ and the certainty of upcoming rewards^100,101^. Our finding that dispositional optimism is foremost associated with the integrity of the CST makes it thus tempting to speculate that optimism is more embodied than we may believe. Could optimism predispose us not only toward expecting good things to happen but also toward motor readiness to go after these rewards? After all, one of the aspects of dispositional optimism bringing an adaptive advantage in life^102^ is its ability to make us effortlessly engage in our goals^103^.

Other individual white matter tracts associated with the “*Optimism*” profile were the dorsal SLF, and the anterior CNG, CC and ATR. The dorsal SLF is essential to the dorsal attention pathway^104–106^ and its integrity relates to measures of sustained goal-directed visuospatial attention in children^107,108^ and adults^109–111^. That optimism and attention deployment are critically linked is demonstrated in various behavioral and neuroimaging studies (^112–115^, for a theoretical account, see^116^). Interestingly, the integrity of the right SLF has further been connected to global visual motion sensitivity^117^, i.e. the ability to perceive and infer the overall movement trend of a multitude of stimuli (e.g. moving dots) with otherwise random motion. In other words, the right SLF supports seeing the forest and not the trees. This holistic perception ability is likely due to the right-side hemispheric lateralization, as discussed above. The overwhelming evidence suggests that the right SLF is involved in the endogenously controlled capacity to actively detect, follow, and respond to relevant (holistic) stimuli over prolonged periods of time. Although the SLF might not be specific to optimism bias, it might maintain it when other mechanisms are in place to generate it, e.g. by supporting long-term pursuit of desirable outcomes^118^.

The remaining white matter tracts whose integrity correlated with the “*Optimism*” component, i.e. the anterior CNG, CC, and ATR, connect brain regions that are part of the anterior midline core of the default mode network (DMN). Notably, the functional connectivity between the core nodes of the DMN positively correlates with the integrity of the CC^119–121^, CNG^122–124^ and the ATR^122,125^. The anterior midline node of the DMN supports internally directed thought and self-referential processing^126,127^, including simulating one’s (mostly optimistically biased) future^128–130^. The integrity of the CC, in particular, has been associated with a higher dispositional optimism^131,132^ and a higher resistance to updating personal beliefs in a pessimistic direction^133,134^. Furthermore, the gray matter endings of the anterior CC, CNG and ATR all overlap in the ventromedial prefrontal cortex (vmPFC;^135–139^). There is highly converging evidence pointing to the involvement of the vmPFC in optimism bias^53,140–144^, and activity in the vmPFC also correlates positively with subjective well-being^145^. Thus, the correlation between the optimism profile and the white matter tracts of vmPFC is not surprising.

In sum, the *“Optimistic*” personality profile correlated with white matter tracts that suggest a motor readiness to act upon the conviction that desired rewards are imminent (CST), a sustained capacity for visually tracking rewards in the environment (SLF) and an intricate network for self-referential processing that helps regulate negative affect and maintain a positive outlook on life (anterior CNG, CC, ATR).

We called the third PCA factor “*Short-term approach behavior*” because impulsivity (indexed by BAS Fun Seeking;^146^), extraversion and agreeableness positively loaded on it. Impulsivity, i.e. the motivation to spontaneously go after novel rewards, is a core part of both extraversion^147,148^ and agreeableness^149^, so their clustering is not surprising. Furthermore, ERQ Suppression loaded negatively, suggesting an inability to suppress impulsive urges. We note that impulsivity is operationally split into motor impulsivity and cognitive impulsivity^150–153^. Motor impulsivity is similar to motor readiness and correlates with CST integrity and excitability (e.g.^154^, see also “Optimism” profile above). By contrast, cognitive impulsivity refers to the inability to compare immediate consequences and the future events with each other and, consequently, the inability to see reasons for delaying immediate satisfaction. The relevant measures on our third PCA factor, namely BAS Fun Seeking^146^, extraversion^147,148^ and agreeableness^149^, measure cognitive impulsivity. Altogether, the “*Short-term approach behavior*” profile correlated negatively with the FA values of the left splenium of CC and left ILF but positively with the RD values of the left splenium and the right UNC/IFOF. FA and RD markers are complementary: low FA and high RD values represent compromised axons (demyelination) while high FA and low RD values represent healthy tracts^77^. Considering this, (impulsive) short-term approach behavior seems to increase as a function of compromised integrity in the left splenium of CC, left ILF and UNC/IFOF. These findings are in line with previous literature on extraversion and compromised posterior CC and IFOF^67,69^ as well as with the literature on BAS Fun-Seeking and compromised left IFOF^55^. Interestingly, individuals with either behavioral or substance addiction show more extensive impairments of the integrity of the CC splenium^155–157^ and ILF^158,159^. These extensive impairments of both CC and ILF are, in turn, inversely related to the levels of self-reported impulsivity^156^. We thus propose that a minimal loss of integrity in these tracts lifts certain barriers and predisposes individuals to be more outgoing and carefree in approaching people and rewards^160,161^, but too extensive a damage to their integrity is a risk factor for addiction and clinical impulsivity.

We termed the fourth PCA factor “*Long-term approach behavior*” due to the high loading of BAS Drive (high-intensity pursuit of desired goals) and BAS Reward Responsiveness (positive affective experience in response to rewards) and, to a lower extent, the contributions of BAS Fun Seeking and Openness. Both BAS Drive and BAS Reward Responsiveness are associated with vigorous pursuit of long-term rewards despite physical or mental challenges^162^, while BAS Fun Seeking points to the spontaneous pursuit of novel rewards^146^. Although, taken in isolation, the three BAS components point to different time windows of pursuing rewards, the combined three components are compatible with pursuing long-term goals. A high BAS Drive safeguards the behavioral maintenance of long-term goals despite obstacles while a high BAS Fun Seeking component (combined with the high BFI Openness) ensures the flexibility and readiness to spontaneously seize unexpected alternative ways of attaining the long-term goal. Finally, a high BAS Reward Responsiveness ensures appropriate positive emotional reactions when attaining each milestone on the way to achieving the final behavioral goal, a process that ensures goal pursuit.

In terms of brain structures, the “*Long-term approach behavior*” component correlated positively with RD values in the right UNC/IFOF: the lower the integrity between the anterior temporal lobes and the frontal cortex, particularly the orbitofrontal cortex^163,164^, the more prominent the long-term approach behavior. It was proposed that the UNC allows temporal lobe-based stimulus associations (e.g. between the physical properties of a stimulus and the emotions associated with it) to modify behavior via interactions with the orbitofrontal cortex^163,164^. These interactions between the temporal lobe and the orbitofrontal cortex would be instrumental in ensuring that stored stimulus associations reflect up-to-date reward and punishment history. Applied to the present data, one may therefore speculate that the successful behavior of keeping eyes on an end goal depends on not always allowing up to date (and likely transitory) reward and punishment information to interfere with the end goal established much earlier on.

### Limitations, strengths and conclusions

We would like to point out that the analyzed sample consisted mostly of females. Given that gender plays a role in brain structure and morphology^165^, future studies should include a balanced sample of males and females. We tried to mitigate these confounding influences by taking age, gender, and total intracranial volume as the covariates of no-interest. Our study has the strength of focusing on personality profiles as opposed to isolated constructs and investigating the white matter correlates of the profiles predictive of subjective well-being. The correlates of an “*Optimistim*” personality profile suggest (a) an intricate network for self-referential processing that helps regulate negative affect and maintain a positive outlook on life, (b) a sustained capacity for visually tracking rewards in the environment and (c) a motor readiness to act upon the conviction that desired rewards are imminent. Correlates of a “*Short-term approach behavior*” profile were indicative of minimal loss of integrity in white matter tracts supportive of lifting certain behavioral barriers, possibly allowing individuals to act more outgoing and carefree in approaching people and rewards. Lastly, a “*Long-term approach behavior*” profile’s association with white matter tracts suggests lowered sensitivity to transient updates of stimulus-based associations of rewards and setbacks, thus facilitating the successful long-term pursuit of goals.

Given that personality plays a crucial role in subjective well-being, identifying the morphological correlates of personality profiles may provide important information for potential therapeutic interventions. For example, an optimistic personality profile was the strongest predictor of subjective well-being both psychologically in terms of the PCA variance and in terms of structural correlates. Some of the putative mechanisms underlying such a profile are domain-general and not specific to optimism per se, e.g., an intact system for sustained attention to holistic stimuli supported by the right SLF. It will be interesting to investigate whether trainings on targeted activities that tap into the functions of the right SLF might transfer to subjective well-being by increasing optimism. Previously, we showed that an attention bias modification training could successfully enhance optimism bias^118^. Future studies can investigate whether such trainings can also boost subjective well-being.

## Methods

### Participants

We recruited 99 healthy candidates with an age criterion between 18 and 40 years (mean age = 24.03 ± 4.39, 62 females). Recruitment was made through emails, flyers and the local participant pool at the University of Bern, Switzerland. Exclusion criteria included self-reported neurological conditions, psychoactive substance usage, left-handedness, and magnetic resonance imaging (MRI) specific problems such as failed image reconstruction and excessive motion artefacts observed on structural scans. For their participation, the students received either course credits or 25 Swiss francs (CHF). Participants gave written informed consent, in accordance with the guidelines of the Declaration of Helsinki. Experimental procedures were approved by the ethics committee of the canton Bern, Switzerland. Five participants were excluded due to incomplete questionnaires. Both DTI and T1-weighted structural scans from all participants qualified visual inspection. Final analysis was conducted on 94 participants.

### Psychological assessment

Using an online portal, all participants completed various psychological questionnaires namely, Comparative Optimism Scale; COS^166^, Satisfaction With Life Scale; SWLS^167,168^, the revised Life Orientation Test; LOT-R^169,170^, Emotion Regulation Questionnaire; ERQ^49,171^, the 10-item Big Five Inventory; BFI^172,173^, Behavioral Inhibition and Behavioral Activation System Scales; BIS/BAS^146,174^. For each participant, the psychological assessment scales amounted to 16 different variables (refer to Table 1). Using PCA allowed us to reduce the multivariate behavioral dataset into fewer meaningful components, thereby enabling a better interpretation of our psychological scales. The resulting personality profiles were then correlated with MRI-based diffusivity measures of FA, AD and RD. The PCA with varimax rotation was performed with XLSTAT^175^. We included the principal components with an eigenvalue > 1. Only Pearson product moment correlation coefficients exceeding 0.4 with a combined alpha level of 0.05 (Bonferroni corrected for multiple testing) were considered for our analytical interpretations.

### Magnetic resonance imaging (MRI) acquisition

MRI measurements were conducted across the study cohort within a 3 T scanner (MAGNETOM Prisma, Siemens, Erlangen, Germany) using a 64-channel head coil, at the Inselspital, University Hospital Bern, Switzerland. The MRI protocol consisted of a 3D magnetization prepared rapid gradient echo (MPRAGE or T1-weighted) sequence with repetition time (TR) = 2300 ms, echo time (TE) = 2.98 ms, inversion time (TI) = 900 ms, flip angle = 9°, matrix size = 160 × 256 × 256 with an isotropic spatial resolution = 1mm^3^. 2D DTI data were acquired consisting of 12 unweighted (b = 0 s/mm^2^) and 90 diffusion-weighted (b = 1000 s/mm^2^) images, with TR/TE = 3200 ms/69 ms, flip angle = 90°, in-plane matrix size = 128 × 128 with 27 axial slices (thickness = 4 mm, spacing between slices = 5.2 mm) and an in-plane resolution of 1.7 × 1.7 mm^2^.

### Image processing

DICOM images from the MRI were converted to NIfTI format using conversion software; dcm2niix (https://github.com/rordenlab/dcm2niix/releases). Computational image processing included tract-based spatial statistics; TBSS^176^ for DTI images. The processes were carried out using FSL6.0.2 (https://fsl.fmrib.ox.ac.uk/fsl/fslwiki) in a MATLAB 2017b environment (developed by The MathWorks, Inc., Natick, United States) utilizing the cluster service UBELIX (https://ubelix.unibe.ch/) from the University of Bern, Switzerland. ‘fsleyes’ from FSL, served as the viewing software for qualitative assessment of the images.

### Tract-based spatial statistics (TBSS)

TBSS performed on DTI data is a commonly used voxel-based statistical framework for understanding white matter integrity^76,177^. Frequently used DTI-derived parameters (from diffusivities: parallel component λ_1_ and perpendicular components λ_2_, λ_3_;^77^) include (a) FA: a measure that could explain the directionality strength of local white matter tracts, (b) AD: a diffusion measure of water molecules diffusing parallel to the axonal tracts, (c) RD: a mean diffusion coefficient of water molecules diffusing orthogonal to the axonal tracts. These diffusivity measures independently should provide complementary information about white matter pathology. For example, FA and AD, on one hand, and RD values, on the other hand, represent the degree of water diffusion parallel and perpendicular to the axonal wall, respectively^77^. As such, these markers are complementary: low FA and AD values and high RD values represent compromised axons (demyelination) while high FA and AD values and low RD values represent healthy tracts. TBSS^76^, the voxel-based approach used in this paper, utilizes extracted and skeletonized white matter tracts in the FA images of all participants. TBSS has been introduced to overcome the problems associated with the use of standard registration algorithms in other voxel-based approaches^177^.

We used the FSL software (http://fsl.fmrib.ox.ac.uk/fsl/fslwiki) for the preprocessing prior to TBSS. First, eddy correction (eddy_correct) was applied on DTI images to address eddy current-induced distortions and motion artefacts. After this step, the DTI images were processed with the FMRIB Software Library’s diffusion toolbox (DTIFIT;^178,179^), which fits a diffusion tensor model at each voxel and generates three eigenvalues (λ_1_, λ_2_, λ_3_). Fractional anisotropy (FA = $\surd \frac{1}{2}\frac{\sqrt{{(\uplambda 1-\uplambda 2)}^{2}+(\uplambda 2-{\uplambda 3)}^{2}+({\uplambda 3-\uplambda 1)}^{2}} }{\left({\uplambda 1}^{2}+{\uplambda 3}^{2}+{\uplambda 3}^{2}\right)}$), axial diffusivity (AD = λ_1_) and radial diffusivity (RD = $\frac{\uplambda 1+\uplambda 2}{2}$) maps were hence generated.

Details of the underlying processes in the TBSS analysis stream are extensively covered in the literature^180–183^. In brief, for the FA maps, firstly a preprocessing erosion step was applied to remove brain edge artefacts and nullify intensities of end slices, to reduce false positives from tensor fitting. Later, FA maps across the participants were non-linearly registered to a standard template in MNI space (FMRIB58_FA template). Following registration, all FA maps were transformed to the standard MNI space with an isotropic voxel size of 1mm^3^, by applying the registration warps to individual images. The standardized maps were merged into a single 4D image, mean FA and its subsequent skeletonized version (WM skeleton) were created. The skeleton was restricted to WM with a threshold of FA = 0.2 and standardized FA maps were projected onto the skeleton. Non-FA maps (RD and AD) were also transformed to their respective skeletonized forms by applying the FA-derived registration and projection vectors. Finally, skeletons across the DTI maps were smoothed with an 8 mm FWHM Gaussian kernel. To find the positive and negative associations between the principal components (from PCA) and the DTI maps (FA, AD, and RD), we performed a non-parametric permutation analysis (t-test) using *randomize* (in FSL) with 5000 permutations, with age, gender, and total intracranial volume (TIV) as the covariates of no-interest. Only the clusters surviving a family-wise error (FWE) multiple comparison at *p* < 0.05 are reported.

### Ethics approval

All procedures performed in studies involving human participants were approved by the ethical committee of the University of Bern according to the standards of the Declaration of Helsinki.

### Consent to participate

All participants provided written consent.

## Data Availability

Under the Swiss guidelines of data protection (Ordinance HFV Art. 5), the MRI images generated and analyzed during the current study can be made available from the corresponding author on a case-by-case basis.
